# An *Epichloë festucae* homologue of MOB3, a component of the STRIPAK complex, is required for the establishment of a mutualistic symbiotic interaction with *Lolium perenne*

**DOI:** 10.1111/mpp.12443

**Published:** 2016-08-14

**Authors:** Kimberly A. Green, Yvonne Becker, Helen L. Fitzsimons, Barry Scott

**Affiliations:** ^1^ Institute of Fundamental Sciences, Massey University Palmerston North 4442 New Zealand

**Keywords:** endophyte, *Epichloë festucae*, MobC, STRIPAK, symbiosis

## Abstract

In both *Sordaria macrospora* and *Neurospora crassa*, components of the conserved STRIPAK (striatin‐interacting phosphatase and kinase) complex regulate cell–cell fusion, hyphal network development and fruiting body formation. Interestingly, a number of *Epichloë festucae* genes that are required for hyphal cell–cell fusion, such as *noxA*, *noxR*, *proA*, *mpkA* and *mkkA*, are also required for the establishment of a mutualistic symbiotic interaction with *Lolium perenne*. To determine whether MobC, a homologue of the STRIPAK complex component MOB3 in *S. macrospora* and *N. crassa*, is required for *E. festucae* hyphal fusion and symbiosis, a *mobC* deletion strain was generated. The Δ*mobC* mutant showed reduced rates of hyphal cell–cell fusion, formed intrahyphal hyphae and exhibited enhanced conidiation. Plants infected with Δ*mobC* were severely stunted. Hyphae of Δ*mobC* showed a proliferative pattern of growth within the leaves of *Lolium perenne* with increased colonization of the intercellular spaces and vascular bundles. Although hyphae were still able to form expressoria, structures allowing the colonization of the leaf surface, the frequency of formation was significantly reduced. Collectively, these results show that the STRIPAK component MobC is required for the establishment of a mutualistic symbiotic association between *E. festucae* and *L. perenne*, and plays an accessory role in the regulation of hyphal cell–cell fusion and expressorium development in *E. festucae*.

## Introduction

Filamentous fungi exhibit complex life cycles. Numerous temporal and spatial cellular changes are required for progressive spore germination, septation, cell–cell fusion and the development of sexual and pathogenic structures, such as fruiting bodies and appressoria. The characterization of several protoperithecia (*pro*) mutants in *Sordaria macrospora*, and *pro* homologues in other fungi, has revealed that components of the fungal striatin‐interacting phosphatase and kinase (STRIPAK) complex are required for multiple developmental processes and appear to coordinate cross‐talk between different signalling pathways, indicating that they have evolved diverse regulatory functions (Kück *et al*., 2016).

The STRIPAK complex in *Sordaria* consists of several proteins: the striatin scaffold protein PRO11; the striatin‐interacting protein PRO22; the kinase activator MOB3; the glycosylphosphatidylinisotol (GPI)‐anchored protein GPI1; the serine/threonine phosphatase PP2A subunits A and C; the germinal centre kinases KIN3 and KIN23; the sarcolemmal membrane‐associated protein PRO45; and several other accessory proteins (Bloemendal *et al*., 2012; Frey *et al*., 2015a, 2015b; Nordzieke *et al*., 2015). Mutant analysis of these components and their respective homologues [HAM‐3 (PRO11), HAM‐2 (PRO22), MOB‐3 (MOB3), HAM‐4 (PRO45), and PP2A‐A and PPG‐1 (PP2A subunits)] in *Neurospora crassa* has shown that components of the STRIPAK complex are required for cell–cell fusion and sexual fruiting body development (Bernhards and Pöggeler, 2011; Bloemendal *et al*., 2010, 2012; Dettmann *et al*., 2013; Frey *et al*., 2015a, 2015b; Fu *et al*., 2011; Maerz *et al*., 2009; Nordzieke *et al*., 2015; Pöggeler and Kück, 2004; Read *et al*., 2012; Simonin *et al*., 2010). In addition, PRO22, KIN3 and KIN24 are associated with septation (Bloemendal *et al*., 2010; Frey *et al*., 2015b), and homologues of PRO11 in *Fusarium verticillioides* (Fsr1), *Aspergillus nidulans* (StrA) and *Colletotrichum graminicola* (Str1) are associated with altered radial growth, ascosporogenesis and virulence (Wang *et al*., 2010, 2016; Yamamura and Shim, 2008).

In *Sordaria*, GPI1 localizes to the plasma membrane, PRO45 to the nuclear membrane, PRO45/GPI1 to mitochondria, and KIN3 and KIN24 to septal pores, whereas, in *N. crassa*, HAM‐2 and HAM‐3 localize to the nuclear envelope (Bloemendal *et al*., 2010; Dettmann *et al*., 2013; Frey *et al*., 2015a, 2015b, Nordzieke *et al*., 2015). A correct localization pattern of HAM‐2 and HAM‐3 is required for MAK‐1 [cell wall integrity (CWI) pathway mitogen‐activated protein (MAP) kinase] nuclear accumulation in a MAK‐2 [pheromone response (PR) pathway MAP kinase]‐dependent manner, as MAK‐2 is required for phosphorylation of the STRIPAK component MOB3 (Dettmann *et al*., 2013). These results demonstrate that the STRIPAK complex components exhibit multiple temporal and spatial localization patterns, and play integral roles in transmitting signals from both the CWI and PR MAP kinase pathways, which, in turn, regulate multiple downstream developmental signalling pathways.

*Epichloë festucae* forms mutualistic symbiotic relationships with species of cool‐season grasses, such as *Festuca* and *Lolium*, by both systemic colonization of the intercellular spaces within the host leaves and by colonization of the leaf surface via epiphyllous growth (Christensen *et al*., 2008; Clay and Schardl, 2002; Schardl, 1996). Mutations in genes encoding components of the Nox complex (*noxA* or *noxR*), CWI MAP kinases and scaffold (*mpkA*, *mkkA* and *so*), and the transcription factor *proA*, abolish cell–cell fusion in culture and trigger proliferative pathogenic‐like hyphal growth *in planta* (Becker *et al*., 2015; Charlton *et al*., 2012; Takemoto *et al*., 2006, 2011; Tanaka *et al*., 2006, 2008, 2013). The co‐association of these phenotypes leads to the hypothesis that cell–cell fusion is required for the maintenance of a mutualistic symbiotic interaction of this fungal endophyte with the host grass.

Given that the STRIPAK complex is important for cell–cell fusion in *N. crassa* and *S. macrospora* (Bloemendal *et al*., 2012; Dettmann *et al*., 2013), but has not been investigated in *E. festucae*, we set out to first identify whether the STRIPAK complex members were conserved in *E. festucae* and to test whether the homologue of the STRIPAK complex component MOB3 has a role in *E. festucae* hyphal cell–cell fusion and maintenance of a mutualistic symbiotic interaction with the plant host *Lolium perenne*.

## Results

### *Epichloë festucae* contains homologues of the STRIPAK complex

To determine whether *E. festucae* contains components of the STRIPAK complex, a tblastn search of the genome sequence was carried out using *S. macrospora* PRO11, PRO22, PRO45 and MOB3 as the query sequences. The *E. festucae* homologues identified were aligned with the *S. macrospora* sequences, as well as the corresponding polypeptide sequences from *Fusarium graminearum*, *Magnaporthe oryzae*, *N. crassa* and *Podospora anserina* (Figs S1–S4, see Supporting Information). The *E. festucae* homologues share 70% and 69% identity to *S. macrospora* and *N. crassa* PRO11 and HAM‐3, 61% and 62% identity to PRO22 and HAM‐2, 57% and 51% identity to PRO45 and HAM‐4, and 48% and 51% identity to MOB3 and MOB‐3, respectively. In addition, they contain all of the conserved domains found in these proteins, suggesting that *E. festucae* contains a functional STRIPAK complex. In particular, MobC contains a MOB domain, serine and threonine phosphorylation domains, a Cys2‐His2 Zn^2+^‐binding domain and an SH3‐binding domain, suggesting that MobC participates in protein phosphorylation and protein–protein interactions. In *S. macrospora*, re‐introduction of the N‐terminal MOB3 domain complements the *mob3* mutant defects, whereas introduction of the C‐terminal domain does not (Bernhards and Pöggeler, 2011). These findings may explain the variability in the sequence of the C‐terminus observed between MOB3 homologues.

To investigate the role of the STRIPAK complex in the regulation of *E. festucae* hyphal cell–cell fusion and the interaction of this endophyte with its host *L. perenne*, we deleted the homologue of *mob3*, which we have named *mobC*, by transforming protoplasts of *E. festucae* with a restriction enzyme‐generated linear fragment of pKG4 (Fig. S5a, see Supporting Information). Polymerase chain reaction (PCR) screening of 50 geneticin‐resistant (Gen^R^) transformants identified six (Δ*mobC*#21, Δ*mobC*#28, Δ*mobC*#31, Δ*mobC*#37, Δ*mobC*#38 and Δ*mobC*#40) that produced banding patterns consistent with targeted replacement events (Fig. S5b). Southern analysis of genomic DNA digests from these transformants confirmed that just two (Δ*mobC*#21 and Δ*mobC*#37) were single‐copy ‘clean’ replacements at the *mobC* locus (Fig. S5c). Based on these results, these two strains were selected for further experiments.

### Culture phenotype of Δ*mobC*

**Figure 1:**
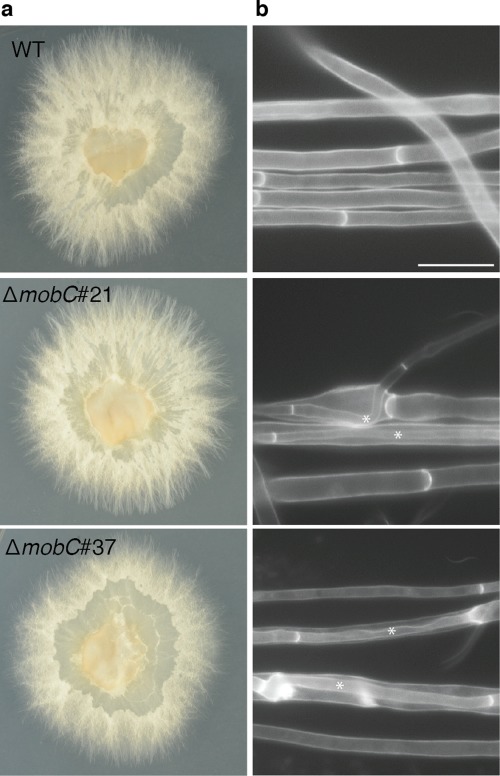
Culture phenotype of *ΔmobC* and wild‐type (WT) strains. (a) Colony morphology of cultures grown on potato dextrose agar for 7 days. (b) Fluorescence images of hyphae grown for 7 days on water agar and stained with calcofluor white. Asterisks indicate intrahyphal hyphae (IHH) in *ΔmobC* mutants. Bar, 10 µm.

**Figure 2:**
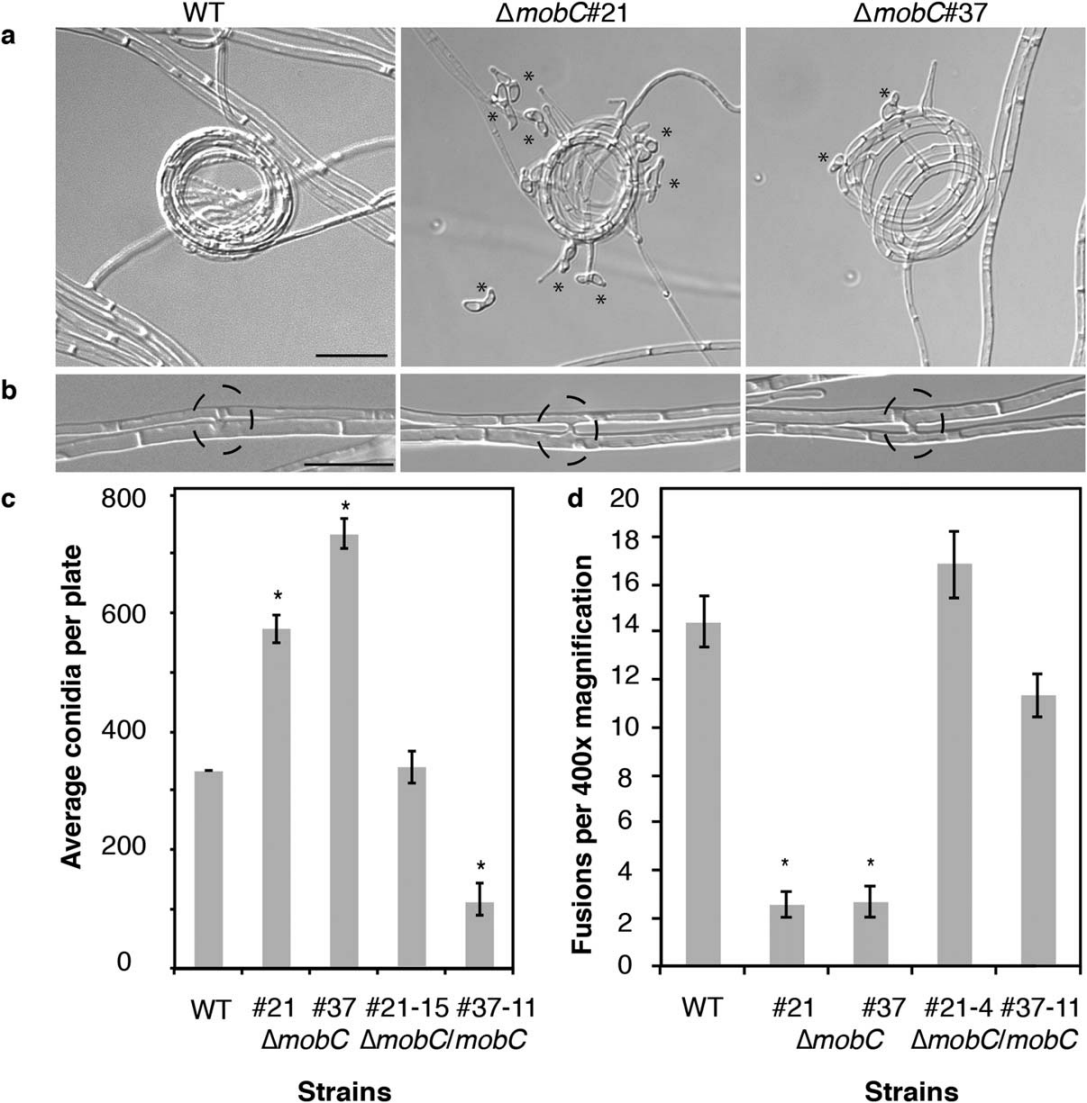
Conidiation and cell–cell fusion phenotype of *ΔmobC*. (a) Differential interference contrast (DIC) images of *ΔmobC* cultures undergoing hyperconidiation on 1.5% water agar after 7 days. Conidia are marked with asterisks. Bar, 20 µm. (b) DIC images of cell–cell fusions in culture as indicated by circles. Bar, 10 µm. (c) Quantification of single colonies recovered from 300 μL of wild‐type (WT), *Δmob*C and *Δmob*C/*mobC* complemented conidia suspensions recovered from a total of 15 cultures grown on potato dextrose agar plates for 7 days. Bars represent mean ± standard error (*n* = 3). Asterisks indicate significant differences from WT as determined by Welch's *t*‐test. (d) Average cell–cell fusions observed per ×40 objective lens magnification field. Bars represent mean ± standard error (*n* = 10). An asterisk indicates significant differences from WT as determined by Welch's *t*‐test.

The culture morphology and radial growth of Δ*mobC* mutants on potato dextrose (PD) were indistinguishable from those of the wild‐type (WT) (Fig. 1a); however, light microscopy revealed that the Δ*mobC* mutants produced intrahyphal hyphae (IHH) (Fig. 1b), a phenotype that has never been observed in WT cultures. In addition, Δ*mobC* mutants showed significantly more conidia than WT (Fig. 2a,c), a phenotype complemented by the re‐introduction of the WT gene (Fig. 2c). Surprisingly, whereas *mob3* deletion mutants in *S. macrospora* and *N. crassa* are completely defective in cell–cell fusion (Bernhards and Pöggeler, 2011; Kück *et al*., 2016; Maerz *et al*., 2009), the *E. festucae* Δ*mobC* strains are still capable of undergoing hyphal cell–cell fusion, albeit at a four‐ to five‐fold lower frequency than that of WT (Fig. 2b,d). Introduction of a WT copy of *mobC* into Δ*mobC* restored the WT fusion phenotype, confirming complementation of this mutant (Figs 2d and S5). These results suggest that MobC plays an accessory role in negatively regulating conidiation and positively regulating hyphal fusion in *E. festucae*.

To test whether deletion of *mobC* affected basal level phosphorylation of the MpkA (CWI) and MpkB (PR) MAP kinases, western blotting was carried out (Fig. S6, see Supporting Information). Two bands were detected in WT, corresponding to phosphorylated MpkA at 47 kDa and phosphorylated MpkB, the Fus3 homologue, at 41 kDa. Although MpkA phosphorylation was not detected in the Δ*mpkA* and Δ*mkkA* CWI kinase mutants (Becker *et al*., 2015), both MpkA and MpkB phosphorylation still occurred in the Δ*mobC*#21 and Δ*mobC*#37 mutants, with bands of similar intensity to that of WT.

### Symbiotic interaction phenotype of Δ*mobC*

**Figure 3:**
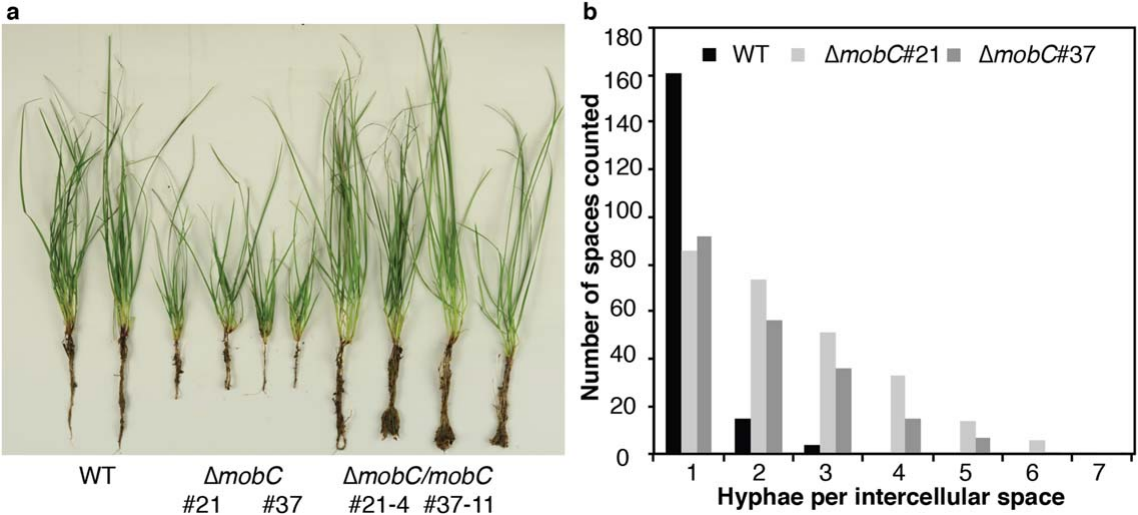
Plant interaction phenotype of *ΔmobC*. (a) Phenotype of infected *Lolium perenne* plants at 10 weeks post‐inoculation. (b) Number of hyphae within the intercellular spaces of the outermost leaf of 10 pseudostem (section of aerial tissue comprising leaf sheath only) cross‐section fields observed at ×100 objective lens magnification using light microscopy. WT, wild‐type.

**Figure 4:**
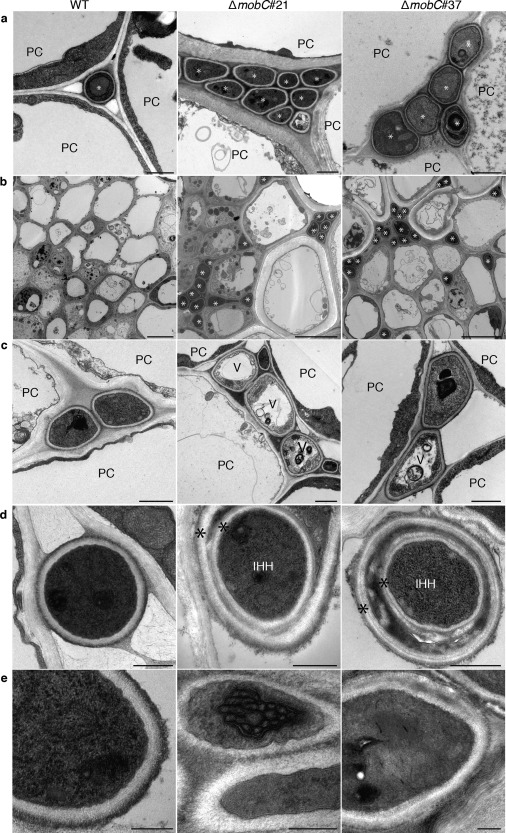
Transmission electron micrographs of *Lolium perenne* pseudostem cross‐sections infected with wild‐type (WT) and *ΔmobC* strains. (a) Hyphae growing within the intercellular spaces between plant cells (PC). Bar, 1 µm. (b) Mutant vascular bundle colonization. Hyphae are marked with asterisks. Bar, 5 µm. (c) Highly vacuolated (V) mutant hyphae within the intercellular spaces. Bar, 1 µm. (d) Mutant intrahyphal hyphae (IHH) formation. Bar, 500 nm. (e) Altered mutant hyphal cell wall. Bar, 200 nm.

**Figure 5:**
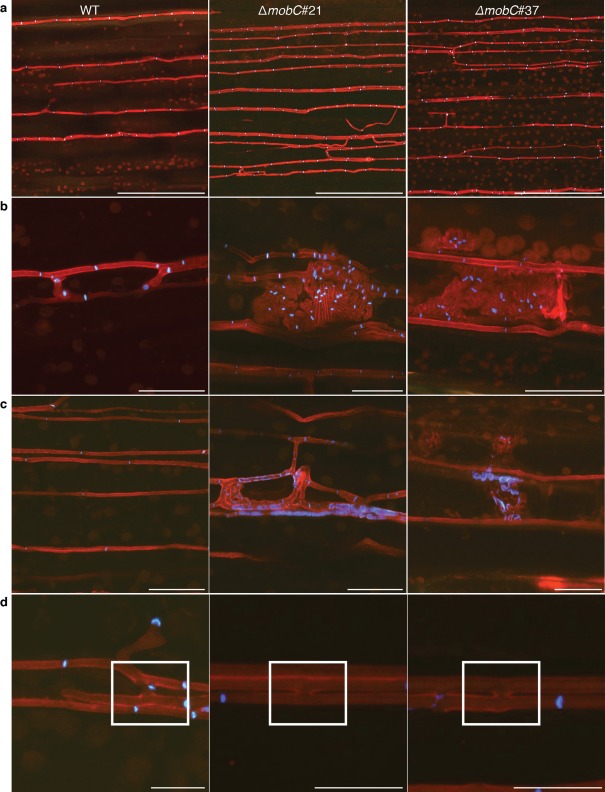
Confocal depth series images of aniline blue/WGA‐AF488 (wheatgerm agglutinin coupled to AlexaFluor488)‐stained *Lolium perenne* leaf sheaths infected with wild‐type (WT) and *ΔmobC* strains. (a) Confocal *z*‐stacks (10 µm) showing increased *ΔmobC* hyphal biomass. Endophytic hyphae show aniline blue fluorescence of β‐glucans captured with red pseudocolour and chitin staining of septa captured by WGA‐AF488 fluorescence with blue pseudocolour. Bar, 100 μm. (b) Aberrant hyphal structures observed in *ΔmobC* associations. Bar, 25 μm. (c) Delocalized WGA‐AF488 fluorescence in *ΔmobC* hyphae. Bar, 25 μm. (d) Hyphal fusions *in planta* as indicated in boxes. Bar, 10 µm.

Given that IHH formation and hyperconidiation are phenotypes shared among many *E. festucae* symbiosis mutants (Becker *et al*., 2015; Tanaka *et al*., 2013), we next tested whether *mobC* is required for the symbiotic interaction of *E. festucae* with *L. perenne*. Plants infected with Δ*mobC* were severely stunted compared with WT‐infected plants (Fig. 3a). Although there were no significant differences in the number of tillers per plant between WT‐ and Δ*mobC*‐infected plants, both tiller length and root length were significantly reduced (Fig. S7, see Supporting Information). Introduction of a WT copy of *mobC* into the Δ*mobC* background rescued the WT symbiotic interaction phenotype (Figs 3a and S7). To evaluate the cellular phenotype of plants infected with Δ*mobC*, we harvested pseudostem tissue samples and examined a range of phenotype parameters by transmission electron microscopy (TEM) (Fig. 4) and confocal laser scanning microscopy (CLSM) (Fig. 5). We observed extensive hyphal colonization in Δ*mobC*‐infected plants, with up to six hyphae per intercellular space in the mesophyll tissue. In contrast, WT associations contained mostly one to two hyphae per intercellular space (Figs 3b and 4a). In addition, the Δ*mobC* hyphae were frequently vacuolated (Fig. 4c); a subset of hyphae contained IHH (Fig. 4d), with the outer cell walls appearing less electron dense (Fig. 4e). Hyphae of Δ*mobC* were also very abundant in the vascular bundle tissue (Fig. 4b), which is seldom, if ever, colonized by WT hyphae. Hyphae in these tissues were electron dense, presumably reflecting the abundant supply of nutrients in these tissues for growth.

The prolific growth of the Δ*mobC* mutant in leaf tissue compared with the more restrictive growth of WT was also evident from CLSM analysis of pseudostem tissue stained with aniline blue (orange/red pseudocolour) and WGA‐AF488 (wheat germ agglutinin coupled to AlexaFluor488, blue pseudocolour), which stain β‐glucan and chitin, respectively (Fig. 5a). Not only were the Δ*mobC* hyphae more abundant than WT hyphae, but they also formed aberrant convoluted hyphal structures comprising many cells, as evident from the many fluorescent septa (Fig. 5b), and exhibited patchy chitin staining (Fig. 5c). Despite these dramatic changes in growth within the plant, Δ*mobC* hyphae were still capable of forming cell–cell fusions *in planta* (Fig. 5d).

### *mobC* plays an accessory role in the regulation of *E. festucae* expressorium formation

**Figure 6:**
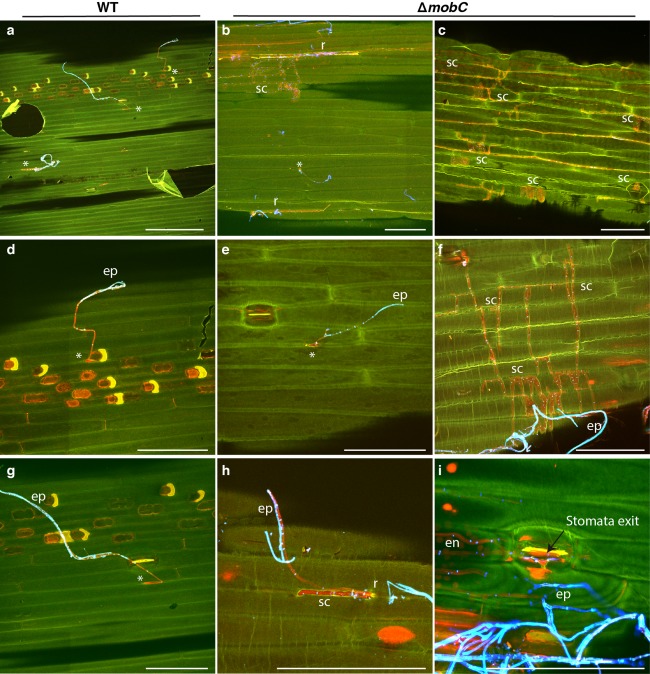
Confocal depth series images of wild‐type (WT) and *ΔmobC* expressoria and subcuticular hyphae within *Lolium perenne* associations. Representative images of the surface of the host pseudostem tissue infected with WT (a, d, g) and *ΔmobC* (b, c, e, f, h, i) strains. Representative images demonstrating the diversity of *ΔmobC* infection phenotypes in which subcuticular hyphae (sc), expressoria (*) and rupture (r) points can be observed (b) or only subcuticular hyphae (c) compared with WT (a). Bar, 200 µm. Zoomed in images of expressoria formation in WT (d, g) and *ΔmobC* (e, f, h, i) strains, with sc aberrant hyphal structures (f), points of rupture (r) (h) and stomata exit (i) in *ΔmobC*. Bar, 100 µm. Fluorescence of endophytic hyphae captured by aniline blue fluorescence of β‐glucans in red pseudocolour. Septa and epiphyllous hyphae (ep) captured by WGA‐AF488 (wheatgerm agglutinin coupled to AlexaFluor488) fluorescence in blue pseudocolour, and soon after exiting the host cuticle layer in green pseudocolour.

Endophytic *E. festucae* hyphae exit the host cuticle layer via an expressorium to form an epiphyllous hyphal network on the surface of the host plant. The formation of these structures requires functional NADPH oxidase NoxA and NoxB complexes (Becker *et al*., 2016). The inability of *nox* mutants to develop expressoria results in the formation of extensive subcuticular growth of hyphae, which eventually breach the surface of the leaves. Given the role of NoxA and NoxB in the development of expressoria in *E. festucae–L. perenne* associations, leaf tissues infected with the Δ*mobC* mutant were examined by CLSM for the formation of these structures (Fig. 6). Although a few expressoria were identified (Fig. 6b,e), the more common phenotype was extensive subcuticular growth of Δ*mobC* (Fig. 6c,f), suggesting that these strains were not fully competent to develop expressoria (Fig. 6). The frequency of expressoria formation in leaf tissue infected with Δ*mobC* was significantly lower than in WT (Fig. S8a, see Supporting Information). Instead, mutant associations had a much greater frequency of subcuticular hyphae (Fig. S8b), which were found to eventually rupture the cuticle of the leaf (Fig. S8c). These results suggest that MobC has an accessory role in regulating the differentiation of *E. festucae* expressoria.

An additional observation was the occasional emergence of hyphae through stomata (Fig. 6i). However, once on the cell surface, the cell walls of mutant hyphae, like WT hyphae, were remodelled with both cell wall and septa binding WGA‐AF488 (captured in blue pseudocolour), whereas only the septa of endophytic hyphae bind WGA‐AF488 (Becker *et al*., 2016).

## Discussion

*Epichloë festucae* NoxA, NoxR, So, ProA, MpkA and MkkA are required for cell–cell fusion in culture and hyphal network formation *in planta*. These findings gave rise to the hypothesis that cell–cell fusion regulates and restricts hyphal growth *in planta* and is required for the establishment of a mutualistic association (Becker *et al*., 2015; Charlton *et al*., 2012; Takemoto *et al*., 2006, 2011; Tanaka *et al*., 2006, 2008, 2013). Here, we show that *E. festucae* MobC, the homologue of the *S. macrospora* STRIPAK complex component MOB3, is essential for the maintenance of a mutualistic symbiotic interaction between *E. festucae* and *L. perenne*, and plays an accessory role in regulating cell–cell fusion and expressoria formation.

In culture, *E. festucae* hyphal strands adhere to one another to form cables, which extend outwards from the colony centre. Cell–cell fusion within these cables occurs by tip‐to‐side fusion events that are easy to observe and quantify using light microscopy (Becker *et al*., 2015). To date, we have been unable to test whether cell–cell fusion mutants of *E. festucae* are also defective in fruiting body development, as the sexual cycle of *E. festucae* is highly complex and only occurs on the plant host and requires a third symbiont, a *Botanophila* fly, to transfer spermatia from a stroma of one mating type to a stroma of the opposite mating type (Bultman & Leuchtmann, 2009). In contrast, *S. macrospora* and *N. crassa* readily form fruiting bodies in culture, enabling phenotype analysis of cell communication mutants in both the vegetative and reproductive stages of development (Engh *et al*., 2010; Fleissner *et al*., 2009; Kück *et al*., 2009; Roca *et al*., 2005; Teichert *et al*., 2014). In *E. festucae*, deletion of *mobC* results in a reduction in cell–cell fusion, but does not completely abolish it, as is the case in *S. macrospora* and *N. crassa* (Bernhards and Pöggeler, 2011; Maerz *et al*., 2009), suggesting that MobC may be less tightly regulated in *E. festucae* than in *S. macrospora* and *N. crassa*, which have different life cycles.

In culture, *E. festucae* conidiation is sparse, whereas Δ*proA*, Δ*noxA*, Δ*mpkA* and Δ*mkkA* mutants show a hyperconidiation phenotype and a loss of cell–cell fusion, suggesting that the regulatory circuits controlling these two phenotypes are linked (Tanaka *et al*., 2013). In addition, Δ*mpkA* and Δ*mkkA* mutants form IHH, structures that have not been observed in the WT strain (Becker *et al*., 2015). Deletion of *mobC* in *E. festucae* also results in an increase in conidiation and IHH formation. In contrast, deletion of *mek‐1* and *mak‐1* and the STRIPAK complex genes *ham‐3* and *mob3* in *N. crassa*, and *ham‐3* homologues *strA* in *A. nidulans* and *str1* in *C. graminicola*, reduces both conidiation and cell–cell fusion, indicating that the regulatory circuits for these developmental processes are ‘wired’ differently in these fungi (Maerz *et al*., 2009; Park *et al*., 2008; Simonin *et al*., 2010; Wang *et al*., 2010, 2016). In addition, no change was observed in the basal phosphorylation levels of MpkA (CWI) and MpkB (PR) kinases in the Δ*mobC* mutant. Given the pleiotropic nature of the Δ*mobC* mutation, it is difficult to determine whether the Δ*mobC* hyperconidiation and IHH formation phenotypes observed are a direct effect of the *mobC* deletion or an indirect effect. Cell–cell fusion is important for colony nutrient transfer, and the reduction or loss of fusion may induce starvation and impair the transport of nutrients to isolated hyphae.

In the grass host, *E. festucae* hyphae absorb nutrients directly from the intercellular spaces of the leaves. Mutations that result in prolific growth *in planta* or disrupt the formation of the hyphal network are likely to trigger a starvation response. This is most probably caused by multiple hyphae per intercellular space requiring and absorbing an excess of nutrients from the apoplast, or from the loss of the hyphal network, impairing nutrient transfer between hyphae. Analysis of the genes up‐ and down‐regulated in the Δ*proA*, Δ*noxA* and Δ*sakA* associations is consistent with this hypothesis (Eaton *et al*., 2015). The genes that are significantly up‐regulated in all three mutant associations include those involved in primary metabolism, peptide and sugar transport, and host cell wall degradation. Key genes that are down‐regulated in this core gene set include genes involved in secondary metabolism and some genes that encode small secreted proteins.

As reported previously for the Δ*proA*, Δ*noxA*, Δ*sakA* and Δ*sidN* mutants (Eaton *et al*., 2010; Johnson *et al*., 2013; Tanaka *et al*., 2006, 2013), Δ*mobC* also forms a pathogenic‐like interaction with *L. perenne*; IHH are frequently observed, as is vascular bundle colonization and breakdown of the hyphal network in the leaves. Although some cell–cell fusions were observed *in planta*, the presence of highly convoluted hyphal structures in the intercellular spaces of Δ*mobC*‐infected plants suggests that cell–cell fusion may also be impaired within the host. Interestingly, Δ*mobC* hyphae within the vascular bundles are electron dense, suggesting that they are much healthier than the mutant hyphae growing in the intercellular spaces. This location‐specific phenotype observed for the Δ*mobC* mutant is consistent with the hypothesis that prolific growth, together with impaired hyphal fusion, triggers a starvation response in hyphae located outside of the nutrient‐rich vascular bundles (Becker *et al*., 2015; Eaton *et al*., 2015).

The homologue of PRO11, *str1*, in *C. graminicola* is required for full virulence in maize and, although Δ*str1* strains produce functional appressoria, infection and further colonization are attenuated (Wang *et al*., 2016). Unlike pathogens, *E. festucae* is not known to colonize leaves by the formation of appressoria‐like structures on the leaf surface. However, a recent study has shown that *E. festucae* does form appressoria‐like structures, named expressoria, that allow endophytic hyphae to breach the cuticle from the inside of the leaf to form a net of epiphyllous hyphae on the surface of the leaf. Deletion of *noxA* and *noxR* abolishes expressoria formation in *E. festucae* and, instead, the mutant hyphae form convoluted branched structures under the surface of the leaf cuticle (Becker *et al*., 2016). Although some WT‐like expressoria structures were observed on the adaxial surface of *L. perenne* leaves infected with the Δ*mobC* mutant, more commonly, subcuticular, highly convoluted structures were observed, suggesting that MobC is important, but not absolutely required, for expressoria development. In this regard, the Δ*mobC* mutant of *E. festucae* is very similar to the *str1* mutant of *C. graminicola* (Wang *et al*., 2016).

In conclusion, *E. festucae* contains highly conserved PRO11, PRO22, PRO45 and MOB3 homologues, suggesting that this fungal endophyte has a functional STRIPAK complex. A phenotype analysis of the *E. festucae* Δ*mobC* mutant showed that MobC is essential for the maintenance of a mutualistic symbiotic interaction between *E. festucae* and *L. perenne*, and plays an accessory role in regulating cell–cell fusion and expressorium development. The Δ*mobC* mutant phenotypes observed are similar to those previously reported for the mutants Δ*mpkA* and Δ*mkkA* in the CWI MAP kinase signalling pathway (Becker *et al*., 2015), suggesting that MobC, MpkA and MkkA are involved in the same signalling pathway. Whether, *E. festucae* MobC is directly linked to the CWI MAP kinase pathway, as proposed for *N. crassa* (Dettmann *et al*., 2013), remains to be tested.

## Experimental Procedures

### Strains and growth conditions

Cultures of *Escherichia coli* were grown overnight in LB (Luria–Bertani) broth or on 1.5% LB agar containing 100 μg/mL ampicillin, as described previously (Miller, 1972). Cultures of *E. festucae* were grown on either 2.4% (w/v) PD, 1.5% water agar plates or in PD broth, as described previously (Moon *et al*., 1999, 2000).

### Plant growth and endophyte inoculation conditions

Endophyte‐free seedlings of perennial ryegrass (*Lolium perenne* cv. Samson) were inoculated with *E. festucae* (Latch and Christensen, 1985). Plants were grown in root trainers in an environmentally controlled growth room at 22°C with a photoperiod of 16 h of light (∼100 μE/m^2^/s) and, at 8 weeks post‐inoculation, were tested for the presence of the endophyte by immunoblotting (Tanaka *et al*., 2005).

### DNA isolation, PCR and sequencing

Plasmid DNA from *Escherichia coli* cultures was extracted using the High Pure Plasmid Isolation Kit (Roche, Basel, Switzerland) according to the manufacturer's instructions. Fungal genomic DNA used for Southern digests was extracted from freeze‐dried mycelium, as described previously (Byrd *et al*., 1990). Standard PCR amplification was performed with *Taq* DNA polymerase (Roche) according to the manufacturer's instructions in a volume of 50 μL. Where proofreading activity was required, Phusion High‐Fidelity DNA Polymerase (Thermo Scientific, Waltham, MA, USA) was used according to the manufacturer's instructions in a volume of 50 μL. Sequencing reactions were performed using the dideoxynucleotide chain termination method with the Big‐Dye™ Terminator Version 3.1 Ready Reaction Cycle Sequencing Kit (Applied BioSystems, Carlsbad, California, USA) and separated using an ABI3730 genetic analyser (Applied BioSystems). Sequence data were then assembled and analysed using MacVector sequence assembly software, version 12.0.5.

### Preparation of deletion and complementation constructs

The lists of all plasmids and primer sequences used to prepare the constructs can be found in Tables S1 and S2 (see Supporting Information).

The *mobC* replacement construct pKG4 was prepared by Gibson Assembly (Gibson *et al*., 2009) using PCR‐amplified pRS426 vector (primers pRS426F and R), 1.2‐kb 5′ (primers KG45 and 46) and 1.5‐kb 3′ (primers KG47 and 48) *mobC* flanking sequences amplified from *E. festucae* genomic Fl1 DNA, and a 1.7‐kb geneticin resistance cassette (primers genF and R), amplified from pII99 plasmid DNA. The *in vitro* recombined DNA mixture was transformed into chemically competent *Escherichia coli* DH5α cells and ampicillin‐resistant transformants were screened using Clonechecker for plasmids with restriction enzyme digest patterns predicted from *in silico* construction of pKG4. The order of the fragments within these clones was verified by DNA sequencing. The *mobC* replacement fragment contained within pKG4 was excised by *Xho*I digestion, gel purified and then transformed into *E. festucae* protoplasts, as described below, using Gen^R^ selection.

The *mobC* complementation construct pKG8 was prepared by Gibson Assembly (Gibson *et al*., 2009) using PCR‐amplified pRS426 vector (primers pRS426F and R) and a 2.7‐kb fragment containing the *mobC* gene (primers KG66 and 67) amplified from *E. festucae* genomic Fl1 DNA. This DNA mixture was transformed into *Escherichia coli* DH5α and pKG8 identified and verified as described above. Plasmid pKG8 was co‐transformed with pSF15.15 into *E. festucae* Δ*mobC* protoplasts as described below using hygromycin‐resistant (Hyg^R^) selection.

### Fungal transformations

*Epichloë festucae* protoplasts were prepared as described previously by Young *et al*. (2005). Protoplasts were transformed with 2–3 μg of linear DNA excised with restriction enzyme or circular plasmid DNA using the method described by Itoh *et al*. (1994). Transformants were selected on regeneration (RG) medium (PD supplemented with 0.8 M sucrose) containing either hygromycin (150 μg/mL) or geneticin (200 μg/mL), and nuclear purified by three rounds of subculture on selection medium.

### DNA hybridization

Following restriction digestion, *E. festucae* genomic DNA was separated by agarose gel electrophoresis, transferred to positively charged nylon membranes (Roche) (Southern, 1975) and fixed by UV light cross‐linking in a Cex‐800 UV light cross‐linker (Ultra‐Lum, Claremont, California, USA) at 254 nm for 2 min. Labelling of DNA probes with digoxigenin‐dUTP (DIG), hybridization and visualization with nitroblue tetrazolium chloride and 5‐bromo‐4‐chloro‐3‐indolyl‐phosphate (NBT/BCIP) were performed using the DIG High Prime DNA Labelling and Detection Starter Kit I (Roche) according to the manufacturer's instructions.

### Western blot analysis

*Epichloë festucae* cultures were grown for 4 days in 50 mL of PD, and 1.5‐g aliquots were weighed into fresh duplicate 50‐mL PD flasks and incubated with shaking (200 rpm) overnight. Samples were washed, flash frozen in liquid nitrogen and freeze‐dried overnight. Protein was then extracted from ground mycelial samples in 1 mL of lysis buffer [50 mm tris(hydroxymethyl)aminomethane (Tris)‐HCl, pH 8, 100 mm NaCl, 10 mm ethylenediaminetetraacetic acid (EDTA), 1 µL/mL IGEPAL CA‐630 (Sigma‐Aldrich, St. Louis, MO, USA), 0.5 mm phenylmethylsulfonylfluoride (PMSF), 2 mm dithiothreitol (DTT) and 10 µL/mL phosphatase inhibitor (Sigma, St. Louis, MO, USA] and centrifuged at 16, 000 g for 15 min at 4°C. Protein samples (70 μg) were separated by sodium dodecylsulfate‐polyacrylamide gel electrophoresis (SDS‐PAGE) [10% (w/v) (Bio‐Rad, Hercules, California, USA)] and transferred to poly(vinylidene difluoride) (PVDF) membranes (Roche). The phosphorylation of MpkA and MpkB was detected using anti‐phospho‐p44/42 MAPK (Erk1/2) antibody (Number 9102; Cell Signalling Technology, Danvers, MA, USA). To check sample loading, the membrane was reprobed with 12G10 anti‐α‐tubulin (Developmental Studies Hybridoma Bank, University of Iowa, Iowa City, IA, USA). Primary antibodies were detected using horse radish peroxidase‐conjugated secondary antibodies and ECL Prime Western Blotting Detection Reagent (GE Healthcare, Amersham, Buckinghamshire, UK).

### Microscopy

Cultures to be analysed by microscopy were inoculated at the edge of a thin layer of water agar (1.5%), layered on top of a glass microscope slide embedded in a layer of water agar (1.5%), and grown for 5–7 days. Square blocks were then extracted and placed onto new slides, covered with a cover slip, and analysed using an Olympus (Shinjuku, Tokyo, Japan) IX71 inverted fluorescence microscope employing filter sets for the capture of differential interference contrast (DIC) or calcofluor (Fluorescent brightener 28, Sigma; concentration, 3 mg/mL) staining. For the quantification of hyphal fusions, 10 fields were examined at ×400 magnification from three independent colonies. For the quantification of conidiation, three PD agar plates, each containing five colonies, were grown at 22°C for 7 days. Conidia were then harvested by scrubbing colonies with 2 mL of sterile water, which was then filtered through glass wool‐packed tips. Suspensions of 300 μL were then spread onto PD agar plates for imaging and quantification.

The growth and morphology of hyphae *in planta* were determined by staining leaves with aniline blue diammonium salt (Sigma) and WGA‐AF488 (Molecular Probes/Invitrogen, Eugene, OR, USA) as follows. Infected pseudostem tissue was sequentially incubated at 4°C in 95% (v/v) ethanol overnight, and then treated with 10% potassium hydroxide for 3 h. The tissue was washed three times in phosphate‐buffered saline (PBS) (pH 7.4) and incubated in staining solution [0.02% aniline blue, 10 ng/mL WGA‐AF488 and 0.02% Tween‐20 in PBS (pH 7.4)] for 5 min, followed by a 30‐min vacuum infiltration step. Images were captured by CLSM using a Leica (Wetzlar, Germany) SP5 DM6000B confocal microscope (488‐nm argon and 561‐nm diode‐pumped solid‐state laser, ×20 or ×40 oil immersion objective, NA = 1.3) (Leica Microsystems). Three photomultiplier tubes (PMTs) were used to capture the emission fluorescence from the dyes, as well as plant autofluorescence. Blue pseudocolour (PMT1, 498–551 nm) was assigned to emission fluorescence from WGA‐AF488 excited with the 488‐nm argon ion laser. Two pseudocolours were assigned to emission fluorescence from aniline blue and plant autofluorescence (PMT2, 571–633 nm, green; PMT3, 661–800 nm, red) as the result of excitation with the 561‐nm DPSS laser (Becker *et al*., 2016). For TEM, pseudostem sections were fixed in 3% glutaraldehyde and 2% formaldehyde in 0.1 m phosphate buffer, pH 7.2, for 1 h, as described previously (Spiers and Hopcroft, 1993). A Philips (Amsterdam, The Netherlands) CM10 transmission electron microscope was used to examine the fixed samples and the images were acquired using a SIS Morada (Müenster Germany) digital camera.

### Bioinformatics analysis

*Epichloë festucae mobC* was identified by tblastn analysis of the *E. festucae* Fl1 (E894) genome (http://csbio-l.csr.uky.edu/ef894-2011) with homologous protein sequences obtained from either the National Center for Biotechnology Information (NCBI) GenBank database (http://www.ncbi.nlm.nih.gov/) or the Broad Institute (http://www.broad.mit.edu). Identity and similarity scores were calculated after ClustalW pairwise alignments of sequences (Thompson *et al*., 1994), using MacVector version 12.0.5, had been performed. The *E. festucae* genome sequence data, as curated by C. L. Schardl at the University of Kentucky, are available at http://www.endophyte.uky.edu (Schardl *et al*., 2013). Sequences for each of the genes analysed in this study are available from that site.

## Supporting information

Additional Supporting Information may be found in the online version of this article at the publisher's website.

**Fig. S1** *Epichloë festucae mobC* gene structure and amino acid sequence alignment. (a) Gene structure showing three exons and two introns of 216, 978, 309, 145 and 32 bp, respectively. Bar, 200 bp. (b) ClustalW alignment of amino acid sequences, with the degree of conserved amino acids indicated by dark–light shading and missing amino acids shown by broken lines. Gene IDs with associated GenBank protein accessions are shown. Protein homologues: *Ef*, *Epichloë festucae* MobC EfM3.028150; *Fg*, *Fusarium graminearum* FGSG_05101.3 (XP_011323597.1); *Nc*, *Neurospora crassa* NCU07674.7/*mob‐3* (XM_957223.3); *Pa*, *Podospora anserina* Pa_6_3550 (XM_001910099.1); *Mo*, *Magnaporthe oryzae* MGG_07095.6 (XM_003715237.1); *Sm*, *Sordaria macrospora mob3* (FN995002.1). Predicted Mob domain (green), serine and threonine phosphorylation sites (orange), Cys2‐His2 Zn^2+^‐binding domain (pink) and SH3‐binding domain (blue) are shown.

**Fig. S2** *Epichloë festucae pro11* homologue gene structure and amino acid sequence alignment. (a) Gene structure showing three exons and two introns of 376, 1871, 258, 84 and 63 bp, respectively. Bar, 200 bp. (b) ClustalW alignment of amino acid sequences with the degree of conserved amino acids indicated by dark–light shading and missing amino acids shown by broken lines. Gene IDs with associated GenBank protein accessions are shown. Protein homologues: *Ef*, *E. festucae* EfM3.057840; *Fg*, *Fusarium graminearum* FGSG_01665.3 (XM_011319188.1); *Nc*, *Neurospora crassa* NCU08741.7/*ham‐3* (XM_958509.2) *Pa*, *Podospora anserina* Pa_6_11770 (XM_001906817.1); *Sm*, *Sordaria macrospora* SMAC_08794/*pro11* (XM_003345392.1). Predicted striatin (red), coiled‐coil (green) and conserved WD40‐binding domains (blue) are shown.

**Fig. S3** *Epichloë festucae pro22* homologue gene structure and amino acid sequence alignment. (a) Gene structure showing six exons and five introns of 755, 146, 89, 1192, 130, 886, 100, 59, 80, 121 and 67 bp, respectively. Bar, 500 bp. (b) ClustalW alignment of amino acid sequences with the degree of conserved amino acids indicated by dark–light shading and missing amino acids shown by broken lines. Gene IDs with associated GenBank protein accessions are shown. Protein homologues: *Ef*, *Epichloë festucae* EfM3.000170; *Fg*, *Fusarium graminearum* FGSG_07159.3 (XM_011328574.1); *Nc*, *Neurospora crassa* NCU03727.7/*ham‐2* (XM_011396271.1); *Pa*, *Podospora anserina* Pa_2_9440 (XM_001911717.1); *Mo*, *Magnaporthe oryzae* MGG_00731.6 (XM_003718223.1); *Sm*, *Sordaria macrospora* SMAC_02580/*pro22* (XM_003352097.1).

**Fig. S4** *Epichloë festucae pro45* homologue gene structure and amino acid sequence alignment. (a) Gene structure showing two exons and one intron of 1029, 1164 and 65 bp, respectively. Bar, 500 bp. (b) ClustalW alignment of amino acid sequences with the degree of conserved amino acids indicated by dark–light shading and missing amino acids shown by broken lines. Gene IDs with associated GenBank protein accessions are shown. Protein homologues: *Ef*, *Epichloë festucae* EfM3.037520; *Fg*, *Fusarium graminearum* FGSG_09221.3 (XM_011330252.1); *Nc*, *Neurospora crassa* NCU00528/*ham‐4* (XM_958987.2); *Pa*, *Podospora anserina* Pa_1_15490 (XM_001906952.1); *Mo*, *Magnaporthe oryzae* MGG_02878.6 (XM_003720805.1); *Sm*, *Sordaria macrospora* SMAC_01224/*pro45* (XM_003352342.1). Predicted forkhead‐associated domain (green) and trans‐membrane helix (blue) are shown.

**Fig. S5** *mobC* deletion and complementation construct design, screening primers and Southern analysis. (a) Schematic diagram of the wild‐type (Fl1) *mobC* genomic locus and linear inserts of the *mobC* deletion construct pKG4 and *mobC* complementation construct pKG8. The regions of recombination are indicated by grey shading. *Pst*I (P) restriction enzyme sites used for Southern analysis and polymerase chain reaction (PCR) primers used for Gibson assembly and knock‐out screening are shown. (b) PCR products of the expected size generated from the primary screen with primers KG37&38 and the secondary screen with primers KG33/34, KG35/36 and KG39/40. (c) Nitroblue tetrazolium chloride and 5‐bromo‐4‐chloro‐3‐indolyl‐phosphate (NBT/BCIP)‐stained Southern blot of *Pst*I (P) genomic DNA digests (1.5 μg) probed with a (DIG)−11‐dUTP‐labelled linear pKG4 fragment purified from a *Xho*I restriction enzyme digest of the plasmid. Fragments of the expected size are shown. WT, wild‐type.

**Fig. S6** Analysis of MpkA and MpkB phosphorylation in Δ*mobC*. Western blot analysis of MpkA and MpkB phosphorylation in wild‐type (WT), TM1066 (TM; a Δ*mkkA* mutant described in Becker *et al*. 2015), Δ*mpkA*, Δ*mkkA*, Δ*mobC#21* and Δ*mobC#37* mutants. Phosphorylated MpkA (47 kDa) and MpkB (41 kDa) were detected using anti‐phospho p42/p44 MAPK antibodies. Tubulin (54 kDa) was used as a loading control and detected using an anti‐α‐tubulin antibody.

**Fig. S7** Quantification of the whole plant phenotype of *Lolium perenne* inoculated with wild‐type (WT), *ΔmobC* and complemented *ΔmobC/mobC* strains. Average tiller length (a), root length (b) and tiller number (c) observed. Bars represent the mean ± standard error (*n =* 7–16). Asterisks indicate significant differences from WT as determined by Welch's *t*‐test.

**Fig. S8** Expressoria phenotype of *ΔmobC*. Number of expressoria (a), subcuticular hyphae (b) and cuticle rupture points (c) observed during associations per leaf at ×200 magnification. All three mutant phenotypes were significantly different from the wild‐type (WT) as determined by a Kruskal–Wallis test and Dunn's multiple comparison test, with *P* < 0.05 (#21) and *P* < 0.01 (#37) for the expressoria phenotype, *P* < 0.0001 (#21 and #37) for the subcuticular hyphae phenotype and *P* < 0.0001 (#21 and #37) for the cuticle rupture phenotype.

**Table S1** Biological material.

**Table S2** Primers used in this study.
