# Supplementary figures and images for: An Epichloë festucae homologue of MOB3, a component of the STRIPAK complex, is required for the establishment of a mutualistic symbiotic interaction with Lolium perenne
Source: Mol Plant Pathol. 2016 Aug 14;17(9):1480–92. doi: 10.1111/mpp.12443 (PMC5132070; doi:10.1111/mpp.12443)

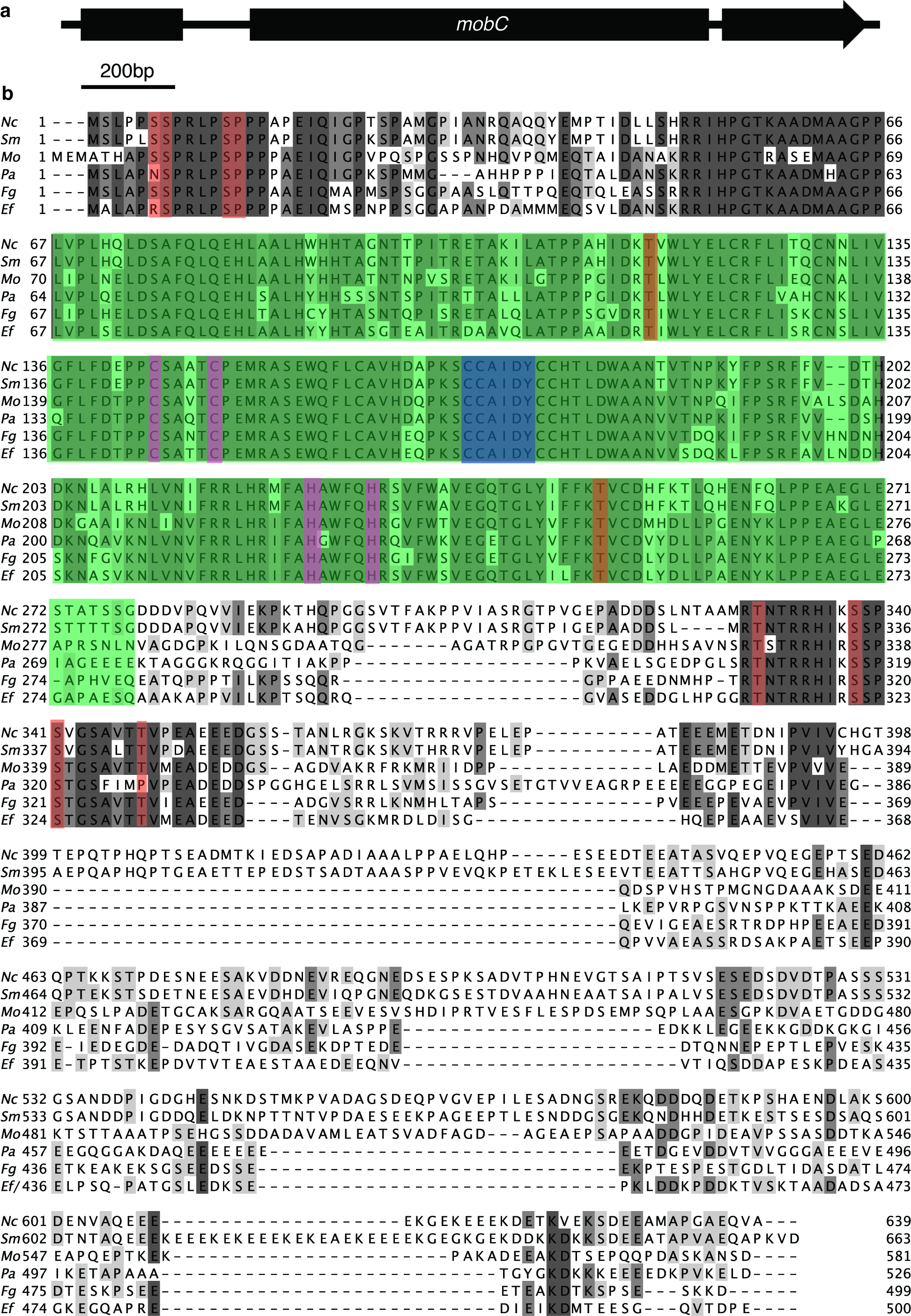

Supplement: Supplementary file 1 — Fig. S1 Epichloë festucae mobC gene structure and amino acid sequence alignment. (a) Gene structure showing three exons and two introns of 216, 978, 309, 145 and 32 bp, respectively. Bar, 200 bp. (b) ClustalW alignment of amino acid sequences, with the degree of conserved amino acids indicated by dark–light shading and missing amino acids shown by broken lines. Gene IDs with associated GenBank protein accessions are shown. Protein homologues: Ef, Epichloë festucae MobC EfM3.028150; Fg, Fusarium graminearum FGSG_05101.3 (XP_011323597.1); Nc, Neurospora crassa NCU07674.7/mob‐3 (XM_957223.3); Pa, Podospora anserina Pa_6_3550 (XM_001910099.1); Mo, Magnaporthe oryzae MGG_07095.6 (XM_003715237.1); Sm, Sordaria macrospora mob3 (FN995002.1). Predicted Mob domain (green), serine and threonine phosphorylation sites (orange), Cys2‐His2 Zn2+‐binding domain (pink) and SH3‐binding domain (blue) are shown. [file MPP-17-1480-s001.tif]

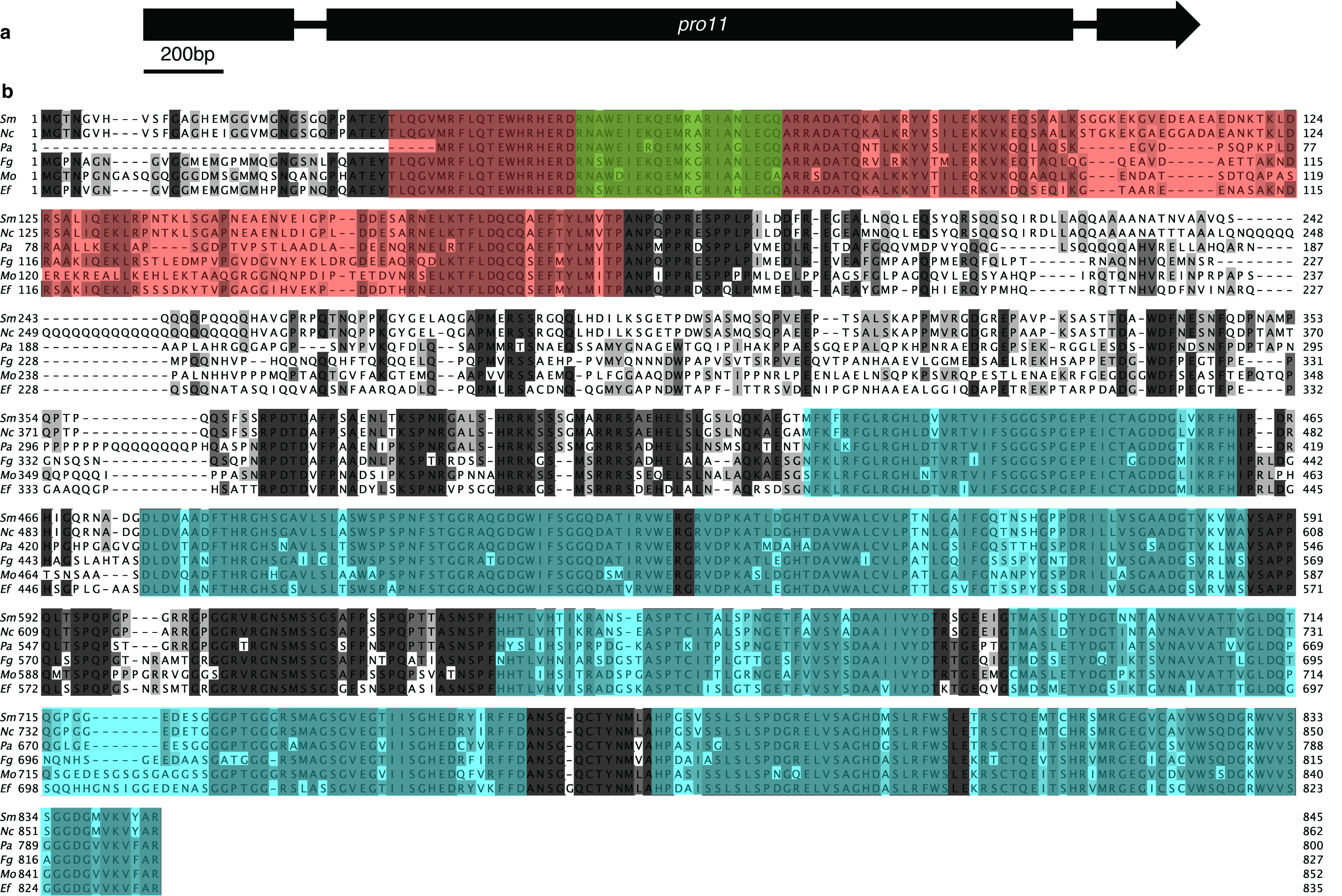

Supplement: Supplementary file 2 — Fig. S2 Epichloë festucae pro11 homologue gene structure and amino acid sequence alignment. (a) Gene structure showing three exons and two introns of 376, 1871, 258, 84 and 63 bp, respectively. Bar, 200 bp. (b) ClustalW alignment of amino acid sequences with the degree of conserved amino acids indicated by dark–light shading and missing amino acids shown by broken lines. Gene IDs with associated GenBank protein accessions are shown. Protein homologues: Ef, E. festucae EfM3.057840; Fg, Fusarium graminearum FGSG_01665.3 (XM_011319188.1); Nc, Neurospora crassa NCU08741.7/ham‐3 (XM_958509.2) Pa, Podospora anserina Pa_6_11770 (XM_001906817.1); Sm, Sordaria macrospora SMAC_08794/pro11 (XM_003345392.1). Predicted striatin (red), coiled‐coil (green) and conserved WD40‐binding domains (blue) are shown. [file MPP-17-1480-s002.tif]

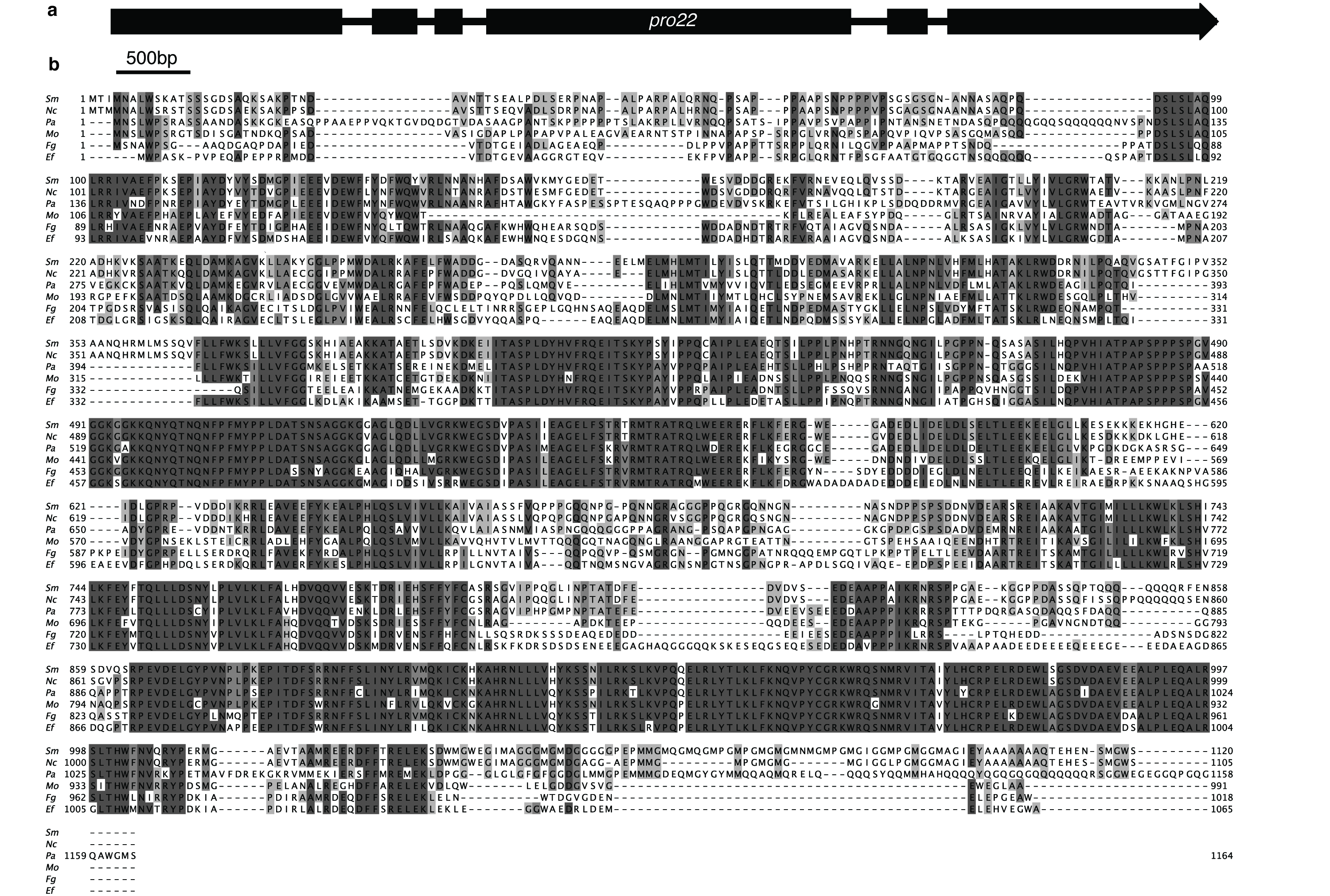

Supplement: Supplementary file 3 — Fig. S3 Epichloë festucae pro22 homologue gene structure and amino acid sequence alignment. (a) Gene structure showing six exons and five introns of 755, 146, 89, 1192, 130, 886, 100, 59, 80, 121 and 67 bp, respectively. Bar, 500 bp. (b) ClustalW alignment of amino acid sequences with the degree of conserved amino acids indicated by dark–light shading and missing amino acids shown by broken lines. Gene IDs with associated GenBank protein accessions are shown. Protein homologues: Ef, Epichloë festucae EfM3.000170; Fg, Fusarium graminearum FGSG_07159.3 (XM_011328574.1); Nc, Neurospora crassa NCU03727.7/ham‐2 (XM_011396271.1); Pa, Podospora anserina Pa_2_9440 (XM_001911717.1); Mo, Magnaporthe oryzae MGG_00731.6 (XM_003718223.1); Sm, Sordaria macrospora SMAC_02580/pro22 (XM_003352097.1). [file MPP-17-1480-s003.tif]

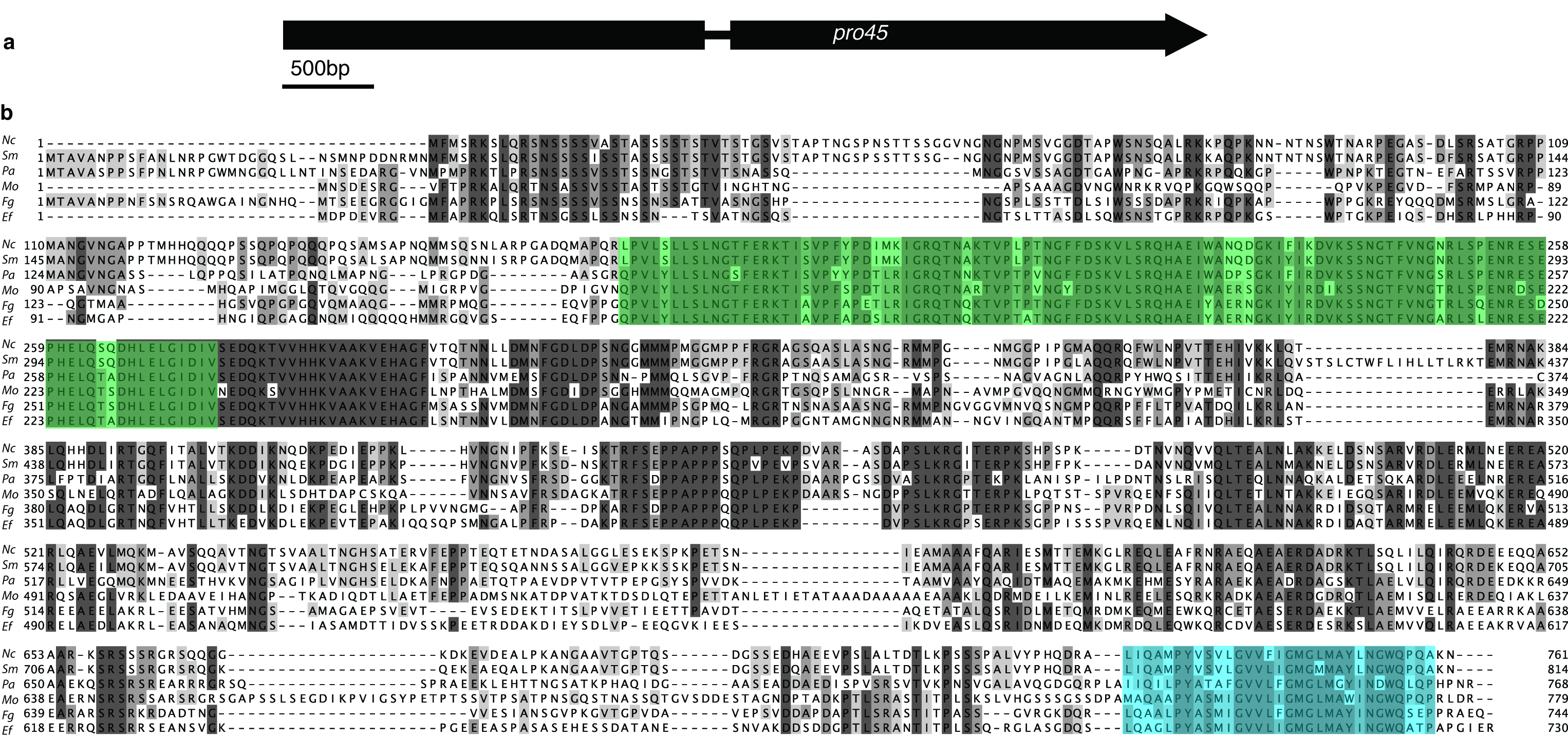

Supplement: Supplementary file 4 — Fig. S4 Epichloë festucae pro45 homologue gene structure and amino acid sequence alignment. (a) Gene structure showing two exons and one intron of 1029, 1164 and 65 bp, respectively. Bar, 500 bp. (b) ClustalW alignment of amino acid sequences with the degree of conserved amino acids indicated by dark–light shading and missing amino acids shown by broken lines. Gene IDs with associated GenBank protein accessions are shown. Protein homologues: Ef, Epichloë festucae EfM3.037520; Fg, Fusarium graminearum FGSG_09221.3 (XM_011330252.1); Nc, Neurospora crassa NCU00528/ham‐4 (XM_958987.2); Pa, Podospora anserina Pa_1_15490 (XM_001906952.1); Mo, Magnaporthe oryzae MGG_02878.6 (XM_003720805.1); Sm, Sordaria macrospora SMAC_01224/pro45 (XM_003352342.1). Predicted forkhead‐associated domain (green) and trans‐membrane helix (blue) are shown. [file MPP-17-1480-s004.tif]

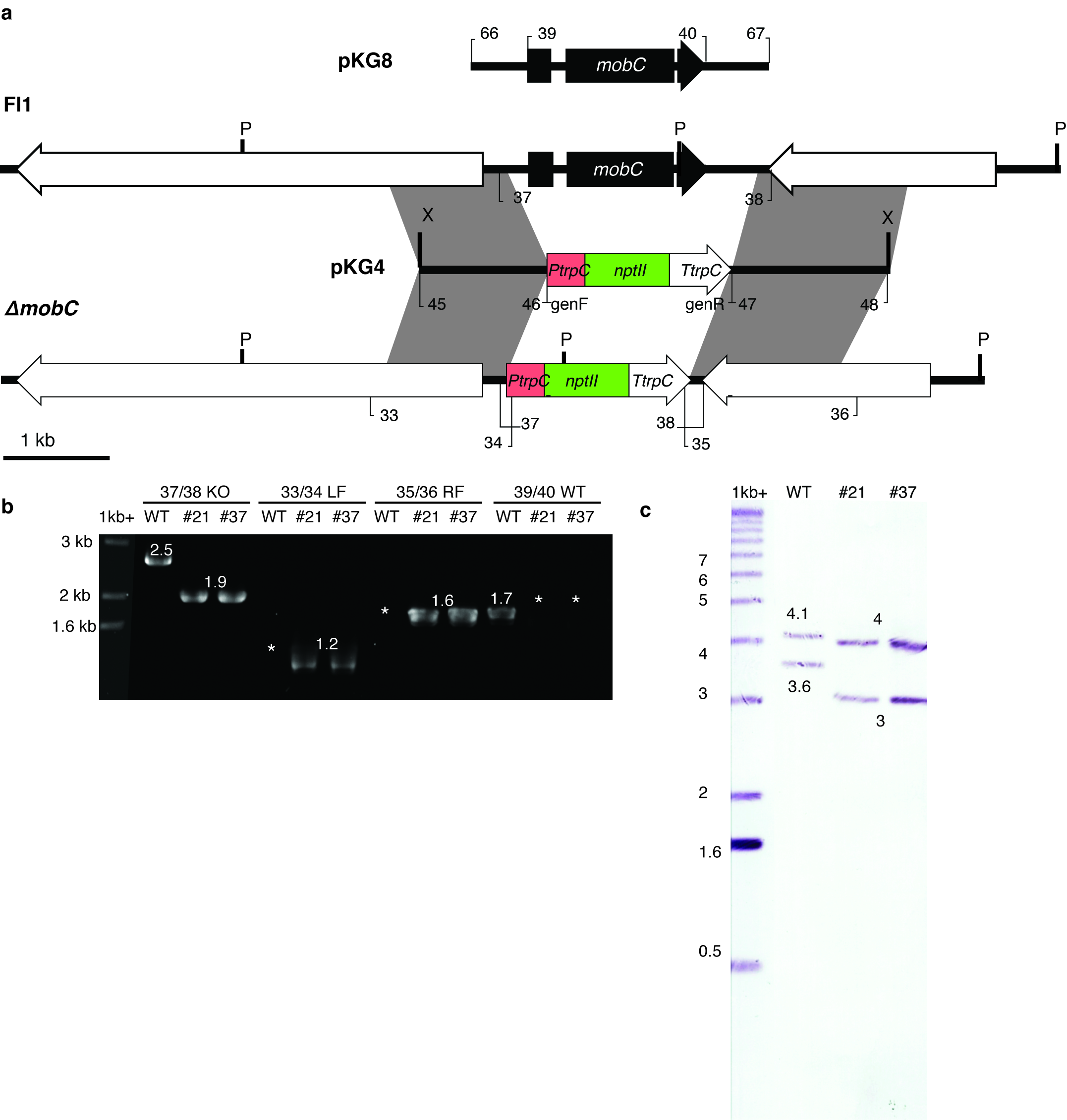

Supplement: Supplementary file 5 — Fig. S5 mobC deletion and complementation construct design, screening primers and Southern analysis. (a) Schematic diagram of the wild‐type (Fl1) mobC genomic locus and linear inserts of the mobC deletion construct pKG4 and mobC complementation construct pKG8. The regions of recombination are indicated by grey shading. PstI (P) restriction enzyme sites used for Southern analysis and polymerase chain reaction (PCR) primers used for Gibson assembly and knock‐out screening are shown. (b) PCR products of the expected size generated from the primary screen with primers KG37&38 and the secondary screen with primers KG33/34, KG35/36 and KG39/40. (c) Nitroblue tetrazolium chloride and 5‐bromo‐4‐chloro‐3‐indolyl‐phosphate (NBT/BCIP)‐stained Southern blot of PstI (P) genomic DNA digests (1.5 μg) probed with a (DIG)−11‐dUTP‐labelled linear pKG4 fragment purified from a XhoI restriction enzyme digest of the plasmid. Fragments of the expected size are shown. WT, wild‐type. [file MPP-17-1480-s005.tif]

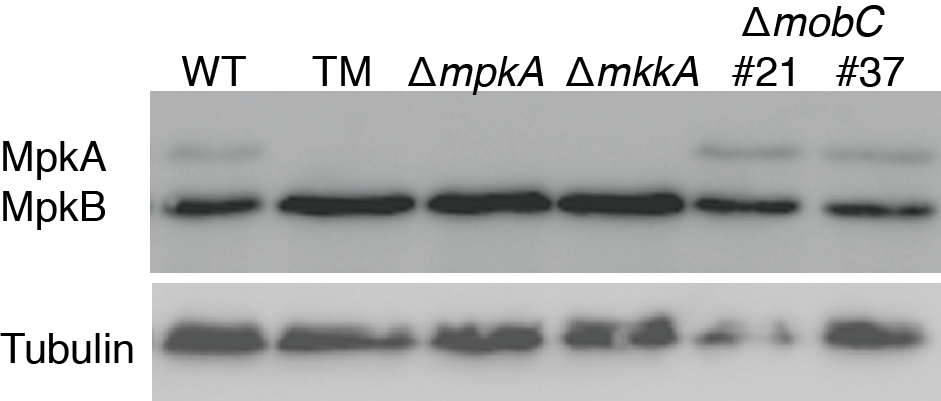

Supplement: Supplementary file 6 — Fig. S6 Analysis of MpkA and MpkB phosphorylation in ΔmobC. Western blot analysis of MpkA and MpkB phosphorylation in wild‐type (WT), TM1066 (TM; a ΔmkkA mutant described in Becker et al. 2015), ΔmpkA, ΔmkkA, ΔmobC#21 and ΔmobC#37 mutants. Phosphorylated MpkA (47 kDa) and MpkB (41 kDa) were detected using anti‐phospho p42/p44 MAPK antibodies. Tubulin (54 kDa) was used as a loading control and detected using an anti‐α‐tubulin antibody. [file MPP-17-1480-s006.tif]

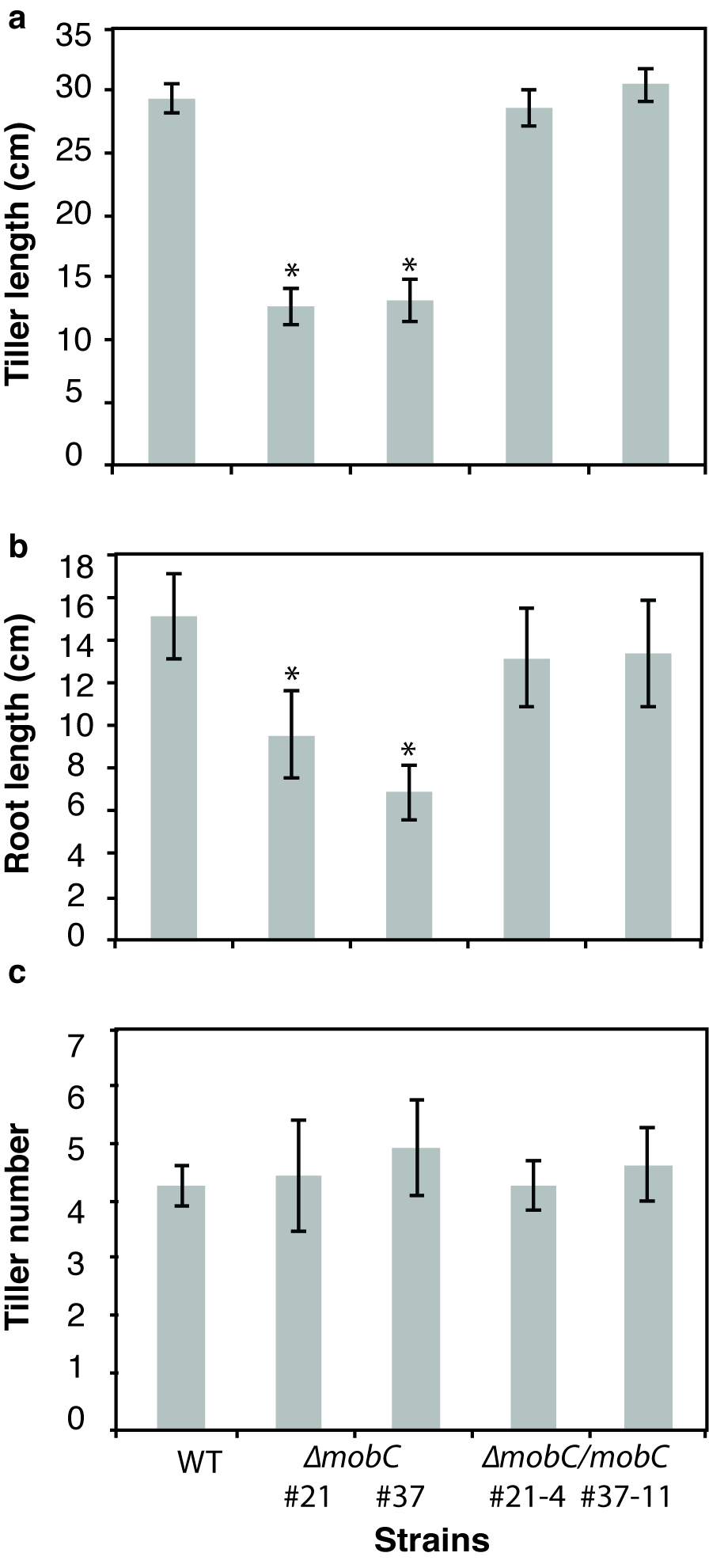

Supplement: Supplementary file 7 — Fig. S7 Quantification of the whole plant phenotype of Lolium perenne inoculated with wild‐type (WT), ΔmobC and complemented ΔmobC/mobC strains. Average tiller length (a), root length (b) and tiller number (c) observed. Bars represent the mean ± standard error (n = 7–16). Asterisks indicate significant differences from WT as determined by Welch's t‐test. [file MPP-17-1480-s007.tif]

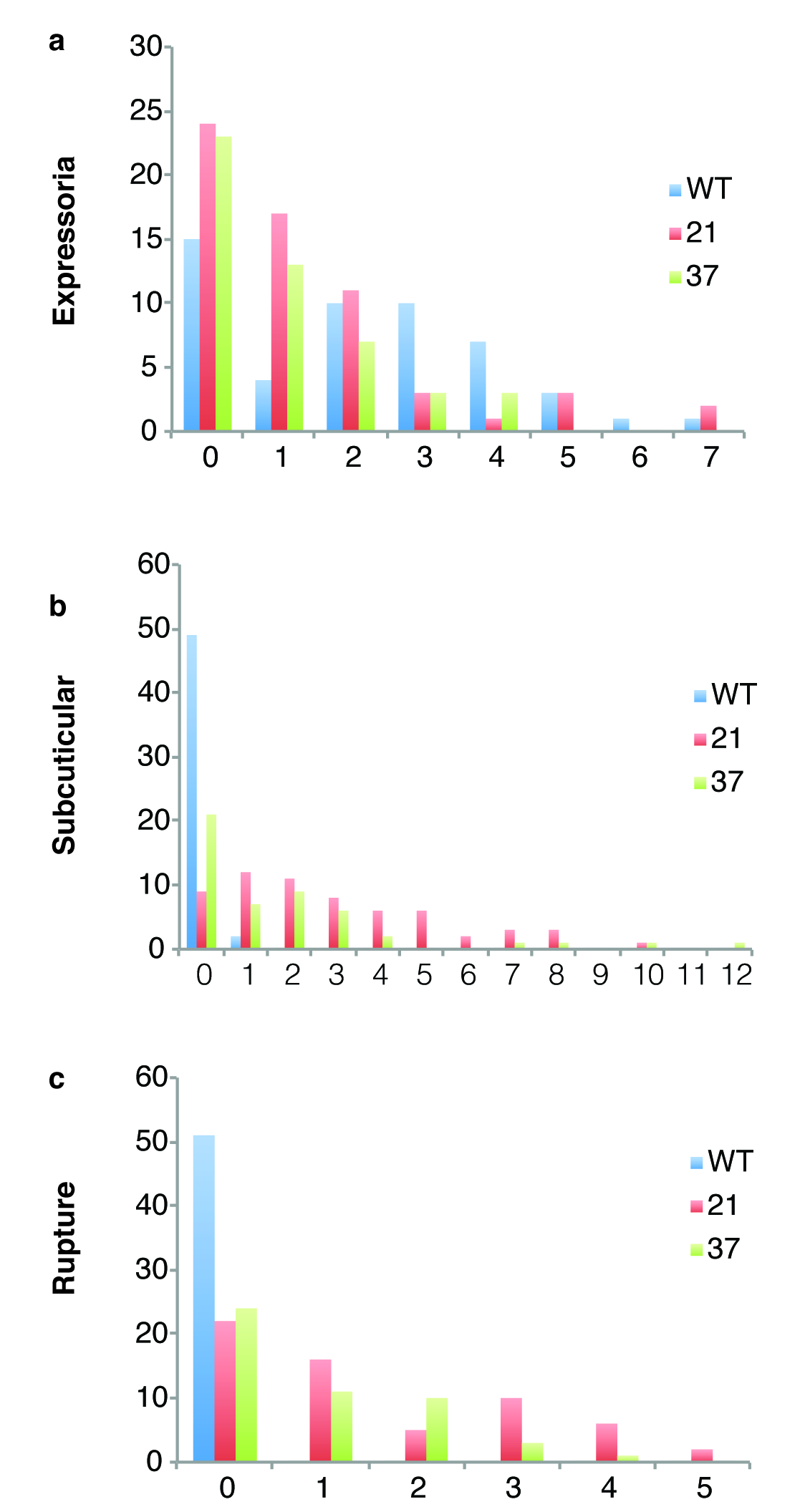

Supplement: Supplementary file 8 — Fig. S8 Expressoria phenotype of ΔmobC. Number of expressoria (a), subcuticular hyphae (b) and cuticle rupture points (c) observed during associations per leaf at ×200 magnification. All three mutant phenotypes were significantly different from the wild‐type (WT) as determined by a Kruskal–Wallis test and Dunn's multiple comparison test, with P < 0.05 (#21) and P < 0.01 (#37) for the expressoria phenotype, P < 0.0001 (#21 and #37) for the subcuticular hyphae phenotype and P < 0.0001 (#21 and #37) for the cuticle rupture phenotype. [file MPP-17-1480-s008.tif]
